# Hyaluronic Acid in Immune Response

**DOI:** 10.3390/biom15071008

**Published:** 2025-07-14

**Authors:** Lech Chrostek, Bogdan Cylwik

**Affiliations:** 1 Department of Biochemical Diagnostics, Medical University of Bialystok, 15-274 Bialystok, Poland; 2 Department of Paediatric Laboratory Diagnostics, Medical University of Bialystok, 15-269 Bialystok, Poland

**Keywords:** hyaluronic acid, immune cells, immune response, signaling pathways

## Abstract

This review summarizes the available evidence on hyaluronic acid’s (HA’s) role in immune response. HA is one of many components in the extracellular matrix that transmits signals from the extracellular microenvironment to cellular effector systems in immune cells. The final effect of these interactions depends on the type of cells and receptors used and the size of HA particles. HA’s activation of intracellular signaling pathways leads to an immune response involving the release of pro- or anti-inflammatory cytokines and chemokines. These play a crucial role in defense mechanisms, such as protecting against pathogens and tissue healing after injuries. HA, as a signaling particle, is also involved in the intensification of the cytokine storm during COVID-19. Multifold increases in HA content in the lungs and the strength of its impact on the immune system define an “HA storm”. The molecular mechanisms involved in inflammation and initiation, including the promotion of cancer, also begin in the microenvironment, and hyaluronic acid is a key element. In this paper, we focus on intra- and intercellular signaling pathways using HA participation rather than injection preparation based on HA use for esthetic treatment.

## 1. Introduction

**Figure 1:**
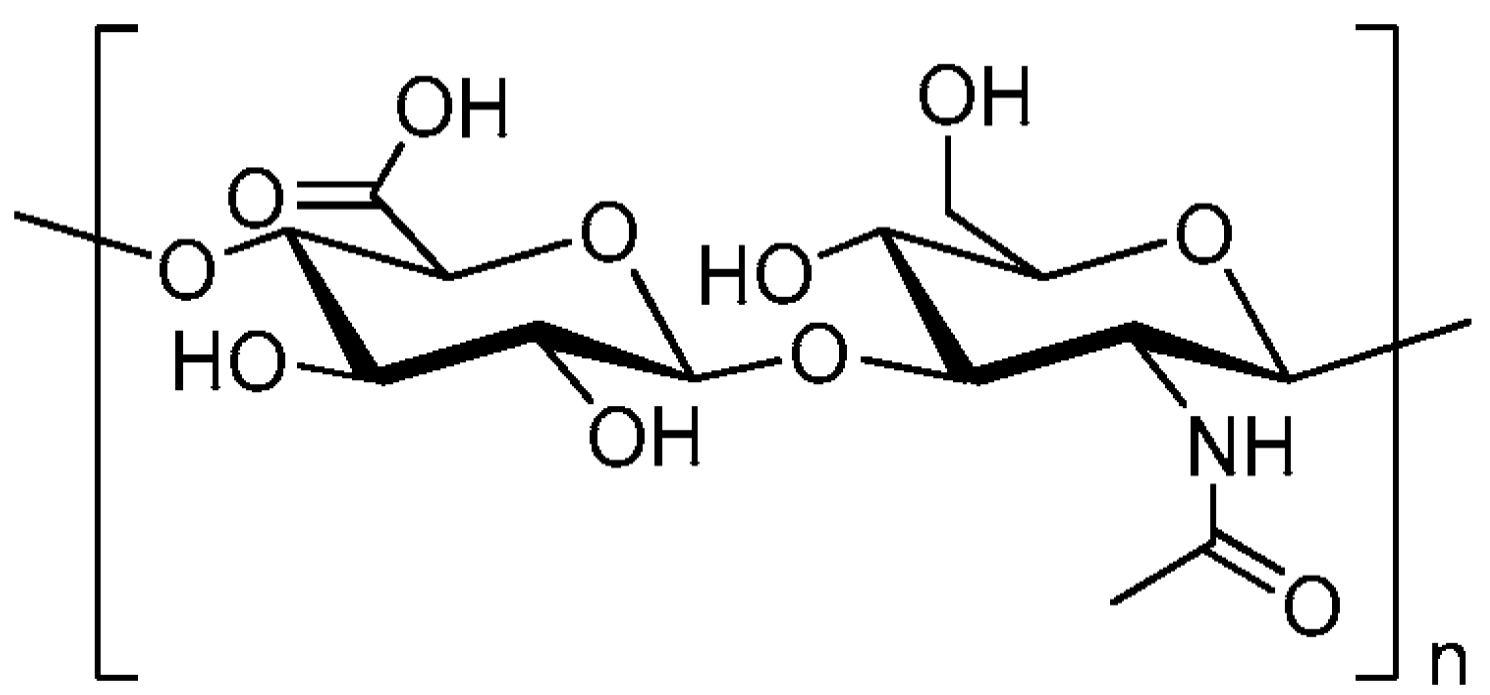
Structure of hyaluronic acid [from our collection].

Hyaluronic acid (HA), also called hyaluronan, is a polysaccharide comprising repeated disaccharide units of D-glucuronic acid and D-N-acetylglucosamine linked by glycosidic bonds [Figure 1] [1]. The number of disaccharide repeats varies from 2000 to 25,000 and correlates to a broad range of molecular weight of 10^5^ to 10^7^ Da [2,3]. At physiological pH, most carboxyl HA groups are deproteinated, resulting in HA that is negatively charged. An atomic force microscopy (AFM) study of HA’s surface can be seen in the work of Cowman and Matsuoka [4].

HA is a major non-sulfated glycosaminoglycan component of the extracellular matrix (ECM) that is attached to other glycosaminoglycans, mainly distributed in the interstitial matrix and the pericellular space [5]. The remaining GAGs are sulfonated (chondroitin, dermatan, and heparan) and bound to core proteins. More than half of HA in the body is found in the skin, and a quarter is in the skeleton and joints together [6]. Different forms of HA are found in human bodies with different molecular sizes. Native HA has a high molecular weight (HMW HA), and its metabolism produces low-molecular-weight molecules (LMW HA) or HA fragments [1]. HA is a key player in immune response, both in terms of innate and adaptive immunity, but its contribution is dependent on the molecular size of HA [2]. LMW HA or its fragments have been shown to induce immune response. Conversely, HMW HA exerts immunosuppressive effects [7,8,9,10,11]. A stimulating or inhibitory effect is transmitted to cellular response via receptors, which have a varied affinity for different forms of HA. For example, if HMW HA binds to the main HA receptor, CD44, it stimulates an immune response (anti-inflammatory activity); if LMW HA binds to this receptor, it has a pro-inflammatory effect [12,13]. The differences in pro- and anti-inflammatory activity depend on the stimulation or inhibition of signaling pathways in immune cells, ultimately resulting in the increased production of pro- or anti-inflammatory cytokines and chemokines [1,5,14].

## 2. HA Metabolism

**Scheme 1:**
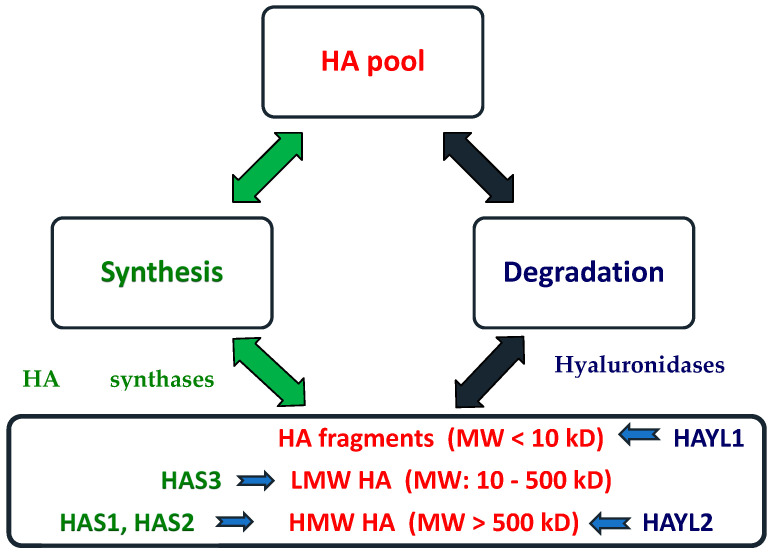
HA balance in the body (from the literature in the text).

HA is synthesized at the cell membrane by three synthases (HAS1, HAS 2, and HAS 3); among these, HAS 1 and HAS 2 are responsible for producing the largest HA molecules. HAS 3, in turn, forms HA with an intermediate molecular size [1,15]. Tissues, plasmatic concentration, and the size of HA in health and diseases are determined by both biosynthesis and the degradation of HA (Scheme 1). HMW HA (>500 kD) takes the form of low-molecular-weight HA (LMW: between 10 and 500 kD), including members of the glycosidase family, called hyaluronidases [HYAL1-4, PH-20, HYALP1] [1,16]. HYAL1 degrades HA into tetra- and hexasaccharides in lysosomes (molecular size < 10 kB), and HYAL2 is responsible for cleaving HMW HA [1,17]. The factors triggering the fragmentation of HMW HA to LMW HA in damaged, inflamed, or infected tissues include reactive oxygen species in the free radical polymerization of HA (ROS) [17,18]. The use of electron paramagnetic spectroscopy identified HA chain scission by hydroxyl radicals generated during the inflammatory stage of arthritis [19]. These radicals are the result of hydrogen atom abstraction from carbohydrates, which generate broad and sharp signals. Broad signals were assigned to HMW HA-derived radicals, while sharp signals corresponded to radicals present either at the ends of the polymer or on LMW fragments. In summary, these radicals generated during the inflammatory stage of arthritis are responsible for the degradation of HA, and this almost certainly causes the loss of synovial fluid viscosity. It may also be the result of enzymatic cutting by hyaluronidases or the synthesis of smaller chains (LMW HA) by HA synthases [1]. When the structure of HA is exposed to pathological conditions (bacterial, viral, or tissue damage), certain types of HA with distinct functional properties have been observed [20,21].

## 3. HA Receptors

It has been widely confirmed that HA plays an important role in innate and adaptive immune response [2,22]. The former involves macrophages, monocytes, natural killer cells, and proteins (enzymes). The adaptive immune system is formed by T and B lymphocytes and antibodies. The ability of HA to undertake immune response is mediated through interactions with surface receptors called hyaladherins [23,24]. There are two main proteins binding HA with receptors via various mechanisms. The first is HA-binding proteins—HABPs—on the CD44 receptor, and the second is TSG-6 [24]. HABPs belong to the link module superfamily, and TSG-6 is a secreted glycoprotein produced by *tumor necrosis factor-stimulated gene-6*. Each of them contains a binding domain: HA-binding domains (HABD_CD44) and Link_TSG6 domains. HABD_CD44 binds HA solely via hydrogen bonds and van der Waals forces, whereas Link_TSG6 primarily binds via ionic and CH-π interactions [24].

Several receptor signals induced by HA play a crucial role in all aspects of immune cell life, including trafficking, adhesion, and proliferation. The activation of signaling pathways triggers immune response or silencing. It is well established that hyaluronan receptors play a distinct role in cytokine production, depending on their molecular size. Low-molecular-weight HA stimulates the binding of monocytes to fibronectin and type 1 collagen by increasing the mRNA expressions of CD40 and CD80 [25].

The ultimate effect of HA binding to a receptor depends on its origin. T-cell proliferation stimulated by endogenous HA is CD44-dependent, but stimulation via exogenous HA mediated by IL-2 is CD44-independent [22].

**Figure 2:**
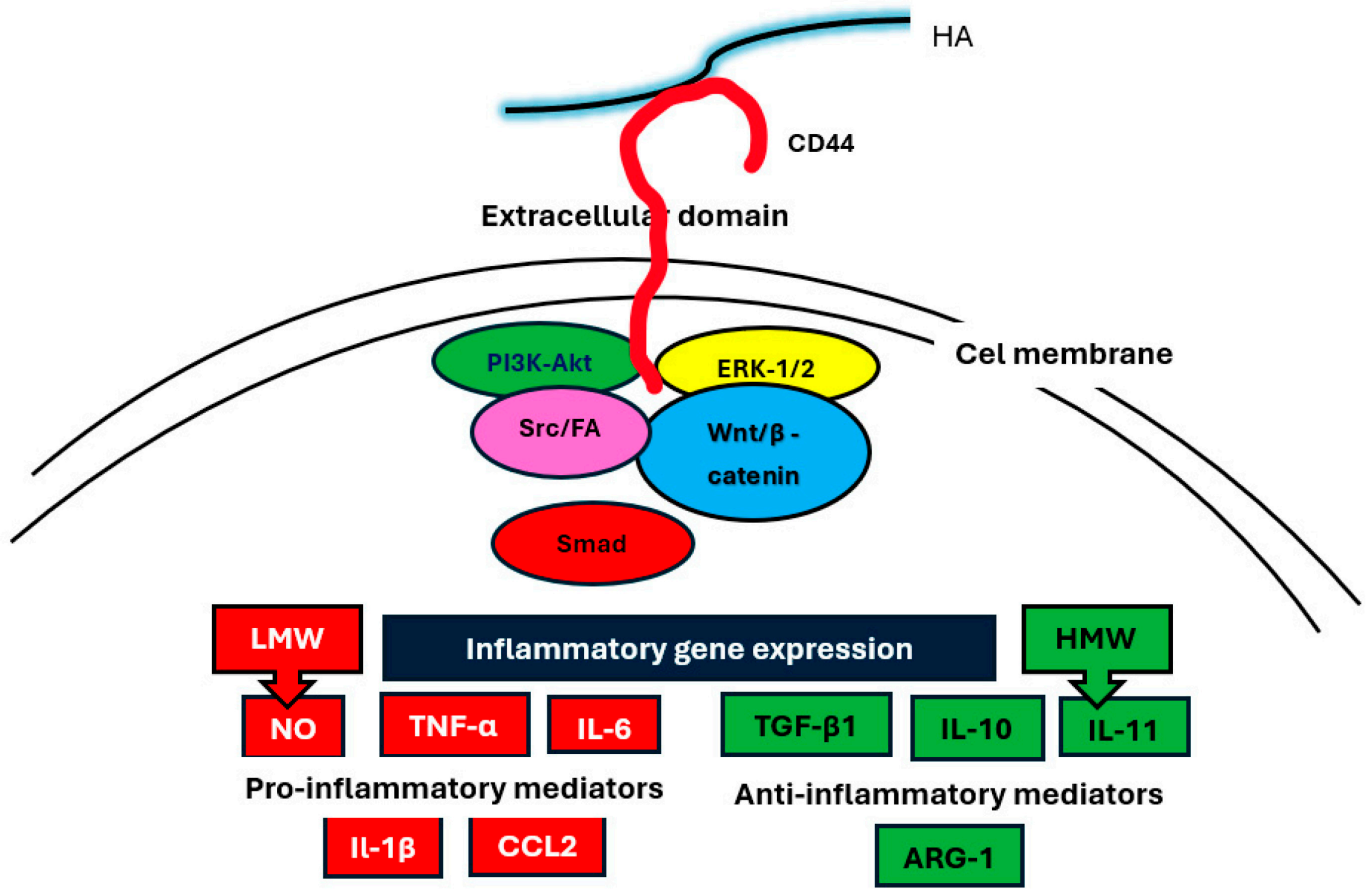
The role of the CD44 receptor in HA-derived signal transduction for effector functions (pro- and anti-inflammatory effects) [5]. ERK-1/2—extracellular signal-regulated kinase 1/2; PI3K-Akt.—phosphatidylinositol 3-kinase; Wnt/β-catenin—Wnt/β-catenin signaling pathway; Src/FAK—tyrosine kinase and focal adhesion kinase pathway; Smad—Smad family protein transducers of TGF-β family growth factors.

HMW HA mainly exerts anti-inflammatory activities through its interactions with the cluster of differentiation-44 (CD44) (Figure 2) [12] and expresses radioprotective effects in the intestine through TLR4 receptors [23]. The pro-inflammatory effects of LMW HA are mediated by its interactions with toll-like receptors 2 and 4 (TLR2 and TLR4), the hyaluronic acid receptor for endocytosis (HARE), and hyaluronan-mediated motility receptor (RHAMM) [5]. Among the HA receptors, CD44, TLR2, and TLR4 are expressed on peripheral blood mononuclear cells (PBMCs), while RHAMM and LYVE-1 (the lymphatic vessel endothelial receptor) are not [26,27]. Small HA fragments are also able to increase their pro-inflammatory cytokines in synovial fibroblasts by activating CD44 receptors (Figure 2) [13]. Therefore, the inhibition of HA degradation reduces pro-inflammatory cytokines in synovial fibroblasts and inflammatory activity in arthritis. HA fragments (LMW HA) have been demonstrated to stimulate human dermal microvascular endothelial cells mediated by TLR4 receptors, suggesting that HMW HA-TLR4 interactions are sensitive markers of the repair process after mechanical injury [28].

CD44 is a type 1 transmembrane glycoprotein expressed by the following immune cells: lymphocytes, monocytes, macrophages, NK cells, and dendrocytes. The interaction between HA and CD44 can be direct or via clustering with other HA receptors [5].

The ability of monocyte CD44 to perform hyaluronan binding is directly attributed to IL-1-alpha, IL-1-beta, IL-3, granulocyte-macrophage colony-stimulating factors (GM-CSFs), TNF-α, and lipopolysaccharide (LPS) [28]. The inhibitors of these interactions are IL-4 and IL-13. It was also demonstrated that T-cell-derived TNF-α mediates HA binding to monocytes induced by cytokines [29].

Clustering CD44 with TLRs activates a signaling cascade with MyD-88/NF-κB, leading to the release of pro-inflammatory cytokines and chemokines. The chemokine family includes macrophage inflammatory protein-1a (MIP-1a), macrophage inflammatory protein-1b (MIP 1b), cytokine responsive gene-2 (Crg-2), monocyte chemoattractant protein-1 (MCP-1), and C-C motif chemokine ligand-5 (CCL5, previously RANTES) [5].

When CD44 is clustered with cellular surface receptors, RHAMM (also known as CD168) activates the signaling pathway of the protein tyrosine kinases family, namely Src and focal adhesion kinases, as well as extracellular signal-regulated (Erk) kinases and protein kinase C [5] RHAMM is an HA receptor expressed on both the surface of cells but also in the cytoplasm, cytoskeleton, and nucleus that lack a link module. RHAMM expression is required on the surface of immune cells for CD 44-mediated cell migration, wound healing, and tumorigenesis [30].

HA can also bind to macrophages and sinusoidal endothelial cells (SECs), which express HARE receptors [31]. The HARE receptor binds together several ligands, including heparin. HARE is half of a full-length Stabilin-2 (STAB2) scavenger receptor. HA binding to the HARE receptor activates ERK signaling pathways with the participation of ERK1/2 kinases. This signaling pathway depends on the size and concentration of HA; oligomeric or small HAs < 40 kDA and those with a larger size do not induce the ERK signaling pathway [32,33].

LYVE-1 is a type 1 integral membrane glycoprotein, which binds soluble and immobilized hyaluronan and plays a key role in leukocyte trafficking in the lymphatic system [24,34,35]. It has also been identified as a lymphatic docking receptor for dendritic cells. LYVE-1 is a low-affinity receptor that distinguishes between different fragments of HA due to its avidity, requiring appropriate receptor self-association and/or HA multimerization [35]. By extension, LYVE-1 receptors are essential for trafficking dendritic cells via the lymphatic system, generating a diverse immune response [36].

Layilin (LAYN) is a novel HA receptor [37]. It is a transmembrane protein homologous with C-type lectins found in the peripheral ruffles of spreading cells. Because LAYN binds to talin’s head domain perimeter and also binds cytoplasmatic β-integrins (vinculin and actin), this protein acts as a linker between the cytoskeleton and the cell membrane.

## 4. HA Activation of Intracellular Signaling Pathways

HA’s interactions with specific receptors activate multiple signaling pathways. The direct interaction between HA and the CD44 receptor leads to the activation of multiple signaling axes, including serin/threonine kinase ERK-1,2 and PI3K-Akt, Wnt/β-catenin, focal adhesion kinase Src/fak, and Smad [5].

Clustering CD44 with TLR4 receptors causes amplified inflammatory responses through NF-kB activation [38] and the MyD-88/NF-πB pathway [39] Thetimulation of TLR-4 receptors by LMW HA also activates the NF-κB signaling pathway [40]. HA binding by HARE receptors initiates an ERK signaling response, dependent on HA size [41]. CD44 clustering with RHAMM mediates the activation of Scr/FAk and ERK kinases via protein kinase C [42], and CD44-HARE activates an ERK signaling pathway [43].

Signaling pathways through LAYN involve the activation of both the RhoA/Rho kinase signal (with the decreased expression of E-cadherin) and NF-πB pathway (with the increased secretion of IL-8 and C5/C5a) [44].

## 5. The Association of HA with the Expression of Cytokines and Chemokines

**Scheme 2:**
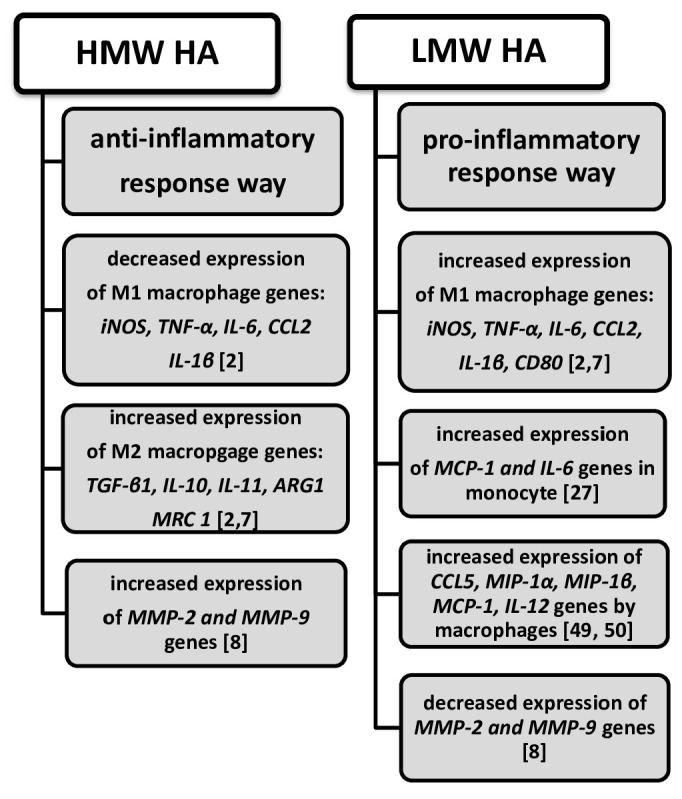
Anti- and pro-inflammatory responses of HMW and LMW HA.

HA manifests an immune response by causing the production of inflammatory mediators; LMW HA promotes the production of inflammatory mediators; and HMW HA inhibits the response (Scheme 2). The activation of an innate immune system (involving monocytes and macrophages) by viral/bacterial infections or tissue damage results in the secretion of inflammatory mediators, such as nitric oxide (NO), tumor necrosis factor-α (TNF-α), and interleukin-6 (IL-6) [11,45]. The key to innate immune system activation is TLR receptors on macrophages, monocytes, and dendritic cells [46,47]. LMW HA directly stimulates the TLR-4 receptor, while HMW HA inhibits macrophage activation [2]. There are two types of macrophages using different methods: classic (M1 macrophage) and alternative (M2 macrophage) activation [2]. M1 macrophage activation increases the expression of pro-inflammatory cytokines and chemokines such as IL-1β, TNF-α, and CCL2 (C-C motif chemokine ligand 2) [2]. The activation of M2 macrophages triggers anti-inflammatory cytokines, such as TGF-β1, IL-10, IL-11, and arginase-1 (ARG1, mannose receptor C-type 1 (MRC 1) [2,7].

The immune response of both CD44 and HA interactions depends not only on the size of HA but also on the cell types and differentiation state of immune cells [48]. Only LMW HA stimulates the production of chemokines (C-C motif chemokine ligand 5 (CCL5, previously RANTES)), macrophage inflammatory protein-1α (MIP-1α), macrophage inflammatory protein-1β (MIP-1β), and IL-12. This occurs in elicited macrophages derived from alveolar and bone marrow but not in resident macrophages [49]. Resident macrophages did not respond to LMW HA. Likewise, peritoneal macrophages and naive T cells bind soluble HA just after their exposure to inflammatory stimuli [20]. The expression of chemokines by macrophages is also induced by HA fragments as small as hexamers [49]. These fragments induce the following chemokine gene family expressions: *MIP-1α, MIP-1β, MCP-1, and CCL5 via CD44 receptors.*

While the HA-mediated signaling pathway causes the release of pro- or anti-inflammatory cytokines, these cytokines both induce and inhibit HA binding to receptors. Some of them, including IL-1α, IL-1β, IL-3, GM-CSF (granulocyte-macrophage stimulating factor), and TNF-α, can immediately induce HA binding to CD44 receptors on monocytes, while others, such as IL-4 and IL-13, inhibit the binding initiated by cytokines [29]. TNF-α plays a key role in this induction because the suppression of its activity by antibodies inhibits the stimulating effect of cytokines when HA binds to monocytes. Some cytokines have a dual effect: IL-10 alone can both stimulate and inhibit the binding of HA to CD44 receptors on monocytes if not previously triggered by IL-2 [29].

The anti-inflammatory effect of some cytokines can also be expressed by inhibited chemokine expressions [50]. IL-10 inhibits the HA-induced expression of MIP-1α and MIP-1β and the cytokine-induced neutrophil chemoattractant (CXCL, previously KC) at both the transcription and protein levels. This proves that HA influences the expression of pro-inflammatory chemokines via cytokines.

## 6. HA in Cytokine Storms During COVID-19

It is unsurprising that HA, as a key component of the extracellular matrix (ECM), is also present within the respiratory airways. In the lungs, HA is found at the surface of alveolar macrophages and type II alveolar epithelial cells [50]. SARS-CoV-2 infection induces cytokine-mediated responses, manifesting as hyperinflation accompanied by rising cytokine levels [11]. Both INF-αβ and INF-γ induce inflammatory infiltration into the lung tissue via monocytes and neutrophils, causing apoptosis of the airway and alveolar epithelial cells. Developing lung injury leads to edema and hypoxia, thus causing acute respiratory distress syndrome (ARDS) [11,51]. Up to 70% of severe COVID-19 patients developed ARDS. On the other hand, the inflammatory cytokines (mainly IL-1β and TNF-α) produced by COVID-19 are also strong inducers of ECM components of metabolism, including HA [52]. The hyperactivation of epithelial cells and macrophages manifests in the form of an increased expression of *hyaluronan synthase 2* (*HAS2*), which causes the hypersecretion of HA in severe COVID-19 cases [53]. Due to *HAS2’s* overexpression (upregulated by IL-1β and TNF-α), HMW-HA synthesis increases exponentially, leading to the accumulation of hyaluronan in alveolar spaces. This increase correlates with the progression of the disease [54]. Because HMW-HA can absorb water over 1000 times its molecular weight, the hyperproduction and accumulation of HA in the extracellular matrix could lead to severe pulmonary edema. In addition, LMW-HA is implicated in the severity of COVID-19 [55,56]. The enhanced synthesis of LMW-HA in ventilator-induced lung injury is promoted via *HAS3* upregulation in fibroblasts. The increased synthesis of HA correlates with the upregulation of genes encoding enzymes involved in hyaluronan metabolism in bronchoalveolar cells [57,58]. The level of this accumulation is so high that it becomes an “HA storm”. This term is legitimate because smaller fragments (LMW-HA) and oligomers formed by the breakdown of HMW-HA increase the release of pro-inflammatory cytokines, thereby intensifying the cytokine storm [59]. The accumulation of HA in the lungs projects a higher HA concentration in the blood, which correlates with the severity of the disease, linking the presence of a cytokine storm directly to patient survival outcomes with COVID-19 [60]. As a result, HA could become a new serum predictive marker for severity and mortality in SARS-CoV-2 infections.

The mechanism of transitioning from mild to severe COVID-19 disease is not fully understood. It has been documented that pre-existing cross-reacting T-cells and antibodies can prevent disease progression [53]. Dysregulation in T and B cell compartments plays an important role in disease severity. A decrease in the CD8^+^ T cell population with elevated levels of IL1B, IL6, and TNF may be one cause. Secondly, severe cases of COVID-19 have a decreased level of T helper 17 cells (TH17) in bronchoalveolar lavage fluid (BALF). Because TH17 cells play an important role in maintaining an antiviral barrier, these cells could be targeted for the treatment and prevention of COVID-19. In addition, dysregulation in B cell compartments should be considered a target for disease exacerbation. The higher frequentation of IgA1-secreting B cells (IgHA1) and a trend of increasing IgG1 in severe cases have been observed and correlated with disease severity. The expressions of *INFA1, metaloproteinases 9MMPs), Mucin 1 (MUC 1), and plasminogen activator inhibitor-1 (PAI-1)* also play an important role in the augmentation of disease severity [53]. Accordingly, severe cases of COVID-19 demonstrate decreased frequencies of *IFNA1*-expressing cells compared to macrophages and epithelial cells (keratin 18^+^ epithelial cell subtypes), an enhanced expression of *MMPs (MMP7 and MMP8)* in epithelial cells without changes to the frequencies of TIMP1^+^- and TIMP2^+^-expressing epithelial cells, an upregulation in the expression of *MUC1* in epithelial cells, and higher frequencies of PAI-1^+^ macrophages.

## 7. HA Provokes Inflammation

ECM components not only create a framework for cells but also facilitate communication routes between GAGs (proteoglycans) and cells via surface receptors. In response to inflammatory signals (infections and tissue damage), the innate immune system responds via leukocyte migration to the site of inflammation. Promoting leukocytes to enter inflamed tissues requires the presence of chemotactic cytokines called chemokines [61]. All these chemokines have GAG-binding domains. In lung diseases (pneumonia and ARDS), one of these chemokines, CXCL8, produced by alveolar macrophages, promotes leukocyte migration and binding to GAGs [62]. One of the components of GAGs to which chemokines bind is hyaluronic acid. As mentioned earlier, HA accumulates in tissues during inflammation as a result of disturbances to its synthesis and degradation due to fluctuations in the activity of enzymes responsible for HA turnover. A rapid increase in the mRNA expression of *HA synthase* in lipopolysaccharide (LPS)-stimulated M1 macrophages during lung inflammation, and its increased expression in bronchoalveolar lavage in patients with asthma has been observed [63,64,65]. Different cells produce HA in response to health and inflammation, including fibroblasts, myofibroblasts, endothelial cells, smooth muscle cells, and type II alveolar epithelial cells (type II ACEs) [66]. However, the mechanism affecting the expression of enzymes involved in HA turnover has not been fully explained. In the case of fibroblasts, the factor increasing HA synthesis is TGFβ which induces *HAS1 and HAS2* expression and reduces *HYL1 and HYL2* expression [67,68]. In turn, TGFβ promotes fibroblast proliferation.

The direct promotion of inflammation by HA is questionable because hyaluronan and hyaluronan fragments without endotoxin do not stimulate TNF-alpha and interleukin-12 or upregulate co-stimulatory molecules in dendritic cells or macrophages [69]. HA accumulation in tissues interacts with immune cells, leading to the expression of pro-inflammatory cytokines and chemokines. A necessary condition for HA binding to surface receptors on monocytes and macrophages is the activation of these cells. CD44 receptors on activated monocytes and peritoneal macrophages do not bind to HA [11]. The factors implicated in the regulation of HA binding to CD44 receptors on immune cells are the granulocyte-macrophage stimulating factor (GM-CSF) and PPARγ in the alveolar space; thus, the capability for HA to bind to CD44 receptors on monocytes and macrophages is required in lung airways [70].

Elevated HA levels in both infarct and remote regions of infarcted hearts coincide with macrophage infiltration after myocardial infarction. HA levels are directed toward a pro-inflammatory state, as confirmed by increased levels of pro-inflammatory cytokines and chemokines such as Il-2, IL-17, and CXCL10 (also known as interferon gamma-induced protein 10—IP-10) [71].

The mechanism triggering inflammation in obesity also begins with an upregulation in the expression of LMW-HA-synthesizing enzymes, such as *hyaluronan synthase-1 (HAS-1)*, in obese adipose tissue [21]. This is accompanied by the overexpression of TLR2 receptors in monocytes and macrophages. The result of the mutual interaction between LMW-HA and TLR-2 receptors is an upregulation in the expression of pro-inflammatory cytokines, such as Il-1β, MCP-1, and IL-8.

## 8. HA Provokes Tumor Growth and Progression

Elevated HA levels have been demonstrated in several types of cancers, including breast, lung, prostate, ovarian, colon, and bladder tumors [3,72,73,74,75,76,77]. Clinical studies and experiments provide evidence that increased levels of HA in tumors result from both the increased expression of HA-synthesizing enzymes and HA-metabolizing enzymes. The overexpression of *HAS1, HAS2, HAS3 HYAL1, and HYAL2* genes causes an overproduction of LMW-HA in the tumor microenvironment, revealing pro-inflammatory and pro-angiogenic properties [72,77]. The enhanced degradation and accumulation of small HA fragments in tumor cells are associated with growth in many ways. Firstly, LMW-HA competes with HMW-HA to bind to the CD44 receptor, inhibiting the Hippo signaling pathway [78,79]. The Hippo signaling network promotes the activation of LATS kinases (large tumor suppressor kinases), which inhibit the activity of transcriptional co-activator proteins such as YAP (yes-associated protein) and TAZ (PDZ-binding motif). Consequently, the *Myc, CycE, and E2F1* genes promote tumor cell growth [79]. The binding of LMW-HA to CD44 receptors abolishes the inhibitory effect of the Hippo signaling pathway on tumor cell growth. Secondly, one of the main factors initiating cancer development is chronic inflammation [80,81,82]. In response to higher levels of HA synthesis and fragments in tumor-associated cells, neutrophils and macrophages produce pro-inflammatory cytokines (IL-1, IL-6, IL-22, and TNF-α) which predispose individuals to cancer mutations. Thirdly, the activation of immune response by hyaluronan triggers the activation of the nuclear factor kappa-light-chain enhancer of activated B cells (NF-kB) [83,84]. All signaling molecules, including TNF-α, IL-1, IL-6, chemokines, MMPs, VEGF (vascular endothelial growth factor), MCSF (macrophage colony-stimulating factor), COX-2 (cyclooxygenase-2), TWIST (Twist-related proteins), and 5-LO (5-lipoxygenase), are regulated by NF-kB [83,84]. It is likely that NF-kB is an essential link between inflammation and cancer. It has been shown that TNF-α upregulation in mouse hepatocytes triggers NF-kB accumulation and TNF-α inhibition, resulting in the apoptosis of transformed hepatocytes and their progress to hepatocellular carcinoma [84]. Various mechanisms have been identified through which NF-kB actively participates in carcinogenesis, involving initiation, promotion, and progression. Thus, NF-kB might participate in colorectal cancer progression by mediating cellular proliferation (through cyclin D1 and cMyc upregulation), angiogenesis (through VEGF, IL-8, and COX2 upregulation), and metastasis (through MMP-9 upregulation) [83]. On the other hand, NF-kB can directly promote cell survival in intestinal cancer by over-producing proteins that inhibit apoptosis, such as Bcl-2 and Bcl-X_L_ [80]. Increasing oxidative stress caused by enhanced levels of reactive oxygen and nitrogen species has a mutagenic effect on DNA, thereby contributing to intestinal tumor initiation. The expression of iNOS is directly activated by TNF-α. NF-kB activation upregulates cytokines and growth factors in gene expression, promoting proliferation, tumor cell survival, and angiogenesis (through IL-6, IL-8, TNF-α, and COX-2 upregulation) [82].

## 9. Conclusions

The binding of hyaluronic acid to receptors is an important step in the initiation of cellular signaling and can induce the expression of genes involved in inflammatory response. Some of these genes have the potential to enact pro-inflammatory and anti-inflammatory responses. We already know that many immunological pathways start from hyaluronic acid and are linked to the stimulus for effector reactions. The key to transmitting a stimulus is the size and concentration of hyaluronic acid used, the interaction between the receptors, and the type of immune cells present. The multifaceted role of hyaluronic acid in immunity causes changes in the microenvironment of immune cells and their function in health and disease. HA, as one of many glycosaminoglycans of the extracellular matrix, not only shapes the microenvironment in which immune cells function but also actively remodels it, influencing the course of inflammation, tissue regeneration during wound healing, and the maintenance of ECM homeostasis in health and disease.

## Data Availability

Not applicable.
